# Development of a video-simulation instrument for assessing cognition in older adults

**DOI:** 10.1186/s12911-017-0557-7

**Published:** 2017-12-06

**Authors:** Edward H. Ip, Ryan Barnard, Sarah A. Marshall, Lingyi Lu, Kaycee Sink, Valerie Wilson, Dana Chamberlain, Stephen R. Rapp

**Affiliations:** 1 Department of Biostatistical Sciences, Wake Forest School of Medicine, Winston-Salem, NC 27157 USA; 2 Department of Gerontology and Geriatric Medicine, Wake Forest School of Medicine, Winston-Salem, USA; 3 Department of Psychiatry and Behavioral Medicine, Wake Forest School of Medicine, Winston-Salem, USA

**Keywords:** Cognitive assessment, Cognitive impairment, Instrumental activity of daily living, Software, Tablet, Simulation

## Abstract

**Background:**

Commonly used methods to assess cognition, such as direct observation, self-report, or neuropsychological testing, have significant limitations. Therefore, a novel tablet computer-based video simulation was created with the goal of being valid, reliable, and easy to administer. The design and implementation of the SIMBAC (Simulation-Based Assessment of Cognition) instrument is described in detail, as well as informatics “lessons learned” during development.

**Results:**

The software emulates 5 common instrumental activities of daily living (IADLs) and scores participants’ performance. The modules were chosen by a panel of geriatricians based on relevance to daily functioning and ability to be modeled electronically, and included facial recognition, pairing faces with the correct names, filling a pillbox, using an automated teller machine (ATM), and automatic renewal of a prescription using a telephone. Software development included three phases 1) a period of initial design and testing (alpha version), 2) pilot study with 10 cognitively normal and 10 cognitively impaired adults over the age of 60 (beta version), and 3) larger validation study with 162 older adults of mixed cognitive status (release version). Results of the pilot study are discussed in the context of refining the instrument; full results of the validation study are reported in a separate article. In both studies, SIMBAC reliably differentiated controls from persons with cognitive impairment, and performance was highly correlated with Mini Mental Status Examination (MMSE) score.

Several informatics challenges emerged during software development, which are broadly relevant to the design and use of electronic assessment tools. Solutions to these issues, such as protection of subject privacy and safeguarding against data loss, are discussed in depth. Collection of fine-grained data (highly detailed information such as time spent reading directions and the number of taps on screen) is also considered.

**Conclusions:**

SIMBAC provides clinicians direct insight into whether subjects can successfully perform selected cognitively intensive activities essential for independent living and advances the field of cognitive assessment. Insight gained from the development process could inform other researchers who seek to develop software tools in health care.

## Background

The proportion of older adults in the United States continues to rise. Cognitive impairment, a condition highly associated with advanced age, is increasingly common (7–8% of older adults) [1]. Cognitive impairment can be difficult to diagnose, and its impact on patient quality of life, functionality, and experience is hard to predict [2]. Accurate identification and measurement of cognitive impairment is essential for diagnosis and treatment planning.

Mild cognitive impairment usually does not interfere with patients’ ability to carry out instrumental activities of daily living (IADLs) [3], which are essential to independent living. Examples of IADLs include taking medication as prescribed, managing money, preparing food, and using the telephone [4]. However, cognitive impairment may still impede IADLs and often worsens over time [5].

Accurate cognitive assessment can predict functional impairment in IADLs. Conversely, detecting limitations in IADLs, especially those that are cognitively demanding, may lead to earlier detection of cognitive impairment. This could be very useful for identifying persons who are safe to continue living independently and those who are likely to require some assistance.

The most commonly used methods of detecting cognitive impairment and functional limitations in IADLs suffer from significant limitations. Direct observation of a patient performing daily tasks by a health care provider is time consuming, expensive, and in some cases difficult to perform. Self-report may be inaccurate, as there is significant potential for bias, such as overstating one’s abilities or poor recall. Report by proxies such as family members may also suffer from bias and proxies may not have adequately observed the patient’s behavior. Neuropsychological testing (NPT) is expensive, time consuming, and puts a heavy burden on the respondent [6]. Abbreviated screeners such as the MMSE [7] and the Clock Drawing Test [8] lack specificity and have ceiling effects [9, 10]. Perhaps the most important limitation of neuropsychological testing is that they are highly inferential in regards to determining what patients can actually do or not do [11].

Although there have been some recent efforts to derive measures of “everyday cognition” or everyday problem solving such as The Everyday Cognitive Battery (ECB) Memory Test [12], they are limited to tasks presentable in paper format [13]. On the other hand, a burgeoning area of interest is the use of electronic media to measure cognition and detect impairment [14]. These assessments have been implemented using desktop computers, handheld tablets, head-mounted displays, and various other projectors and sensors. Perhaps the most straightforward approach has been the adaptation of traditional paper-and-pencil tests to these new formats. For example, Inoue et al. (2011) developed a computerized version of the ADAS-Cog screener [15]. Their tablet-based iteration could test cognition equally as well as the original, but was much faster and didn’t require interpretation by an expert. Still, there are familiar limitations to these adaptations, such as the need to extrapolate real world functioning based on somewhat abstract cognitive tests such as copying of geometric figures.

More recently, researchers have sought to infer cognitive status based on performance in innovative virtual settings and simulations. The test subject is able to interface with a computer-generated situation that is safe and experimentally consistent. Participating in such simulations may be more engaging and enjoyable for participants than traditional assessments. More importantly, simulations of real world activities are more ecologically valid than abstract tests – they can provide direct insight into day-to-day patient functionality.

A recent study exposed healthy elderly, persons with amnestic MCI (Mild Cognitive Impairment), and persons with mild AD (Alzheimer’s Disease) to a virtual simulated fire evacuation drill [16]. Moving from an apartment to the evacuation zone required participants to tap into multiple cognitive domains and respond appropriately. The AD group performed worse than the MCI group, which performed worse than the healthy controls. There was a strong correlation with standard assessments used in the elderly such as the MMSE and Bristol ADL scale. A limitation of this study was the dependence on a relatively complicated setup, including a split-belt treadmill with force plates and curved rear projection screen.

Another study used a virtual supermarket to screen for MCI [17]. Using a tablet, participants navigate the supermarket and procure various items on a list. After all the items are retrieved, the groceries are purchased using a precise amount of bills and coins selected on the screen. This software was initially developed to reinforce or train key cognitive processes such as memory, planning, and attention. Researchers subsequently demonstrated performance in the supermarket correlated highly with established tests of cognition and could correctly identify most persons with MCI. However, this study included only 21 healthy controls and 34 persons with MCI. Additional simulations are undergoing development [18].

Given the limitations of commonly used measures and the promise of modern technology, a novel, interactive video-based simulation administered using computer tablets was developed to fill the gap in cognitive assessment. The SIMBAC (Simulation-Based Assessment of Cognition) program is intended to be low cost, easy to administer, well-tolerated, and ecologically relevant to cognitively demanding IADLs.

Three phases of development - initial design and prototyping, a small pilot study, and a larger validation study - are described, including results from the pilot study and limited preliminary results from the validation study. Preliminary results are limited in scope and include a smaller number of participants. These results are presented in the context of informing development and demonstrating feasibility. Informatics challenges that arose during this period, related to security, portability, scalability, and robustness, and the subsequent lessons learned, are also discussed in depth.

## Implementation

**Fig. 1:**
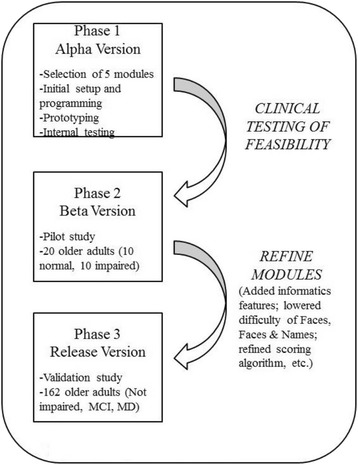
Diagram of developmental phases of SIMBAC (MCI = mild cognitive impairment, MD = mild dementia)

The development process can be divided into 3 phases: initial design and prototyping (alpha version), pilot study (beta version), and validation study (release version) (see Fig. 1). The small pilot study in phase 2 established the feasibility of the assessment tool and identified areas in which the instrument as well as its administration could be improved. Phase 3 included a full-scale validation study with a larger sample of participants and led to the creation of a final version of SIMBAC intended for release.

### Phase 1: Initial development

Three geriatric health care providers (S.R., K.S., V.W.) identified 5 activities that (a) were cognitively demanding, (b) were relevant to everyday life for most older Americans and (c) could be simulated on a computer. They were: recognizing faces, remembering names, filling a pillbox, using an automated teller machine (ATM) and refilling a prescription by phone.

Based on input from the geriatric team, a computer programmer (R.B.) developed a prototype of the 5 SIMBAC modules. The software was written in ActionScript 3 and used the Apache Flex software development kit (SDK). Configurable aspects of the tool engine and individual modules were specified in easily-editable XML files. User-facing strings (e.g. instruction text) were also externalized into XML files. The build and deployment procedure was automated using Apache Maven and Apache Ant. The instrument’s visual assets were variously designed and assembled using Adobe Flash, the GNU Image Manipulation Program (GIMP), and Inkscape. The verbal auditory assets were recorded and edited using Apple Logic Pro.

In the course of designing the instrument, the order and character of each module underwent numerous variations and iterations, and it was often helpful to be able to demonstrate a single module in isolation while gathering feedback. We also observed that, though the content of each module differed in many ways, there were also significant commonalities: each module presented instructions, collected timing and interaction data, and was subject to behavior and content modification at runtime via configuration files.

These observations motivated one of the key design decisions of the instrument’s implementation. The behavior and functionality that was common across all of the modules was implemented in a Task class, with each module being represented by a separate subclass of Task: PhoneTask, PillboxTask, ATMTask, etc. At runtime, an instance of another class, the TaskRunner, runs each module through its complete lifecycle: instantiation, configuration, presentation, result collection, and termination. The order of the modules is defined in an external configuration file, so changing the order of the modules--or using only a subset for demonstration purposes--is as simple as swapping the a few lines of text.

External configuration files also contained the task design for each module, including order of data presentation, duration of stimulus, instruction text, etc. For example, the ATM task externalizes, among other things, the account PIN, the amount of money to withdraw, and which account to withdraw the money from. The instruction text in the configuration files can also reference these settings using string interpolation (e.g. “Your PIN is ${pin}.”). This ensures that the instructions presented to the participant is always consistent with the configured settings since each setting is specified in exactly one location.

#### Facial recognition

**Fig. 2:**
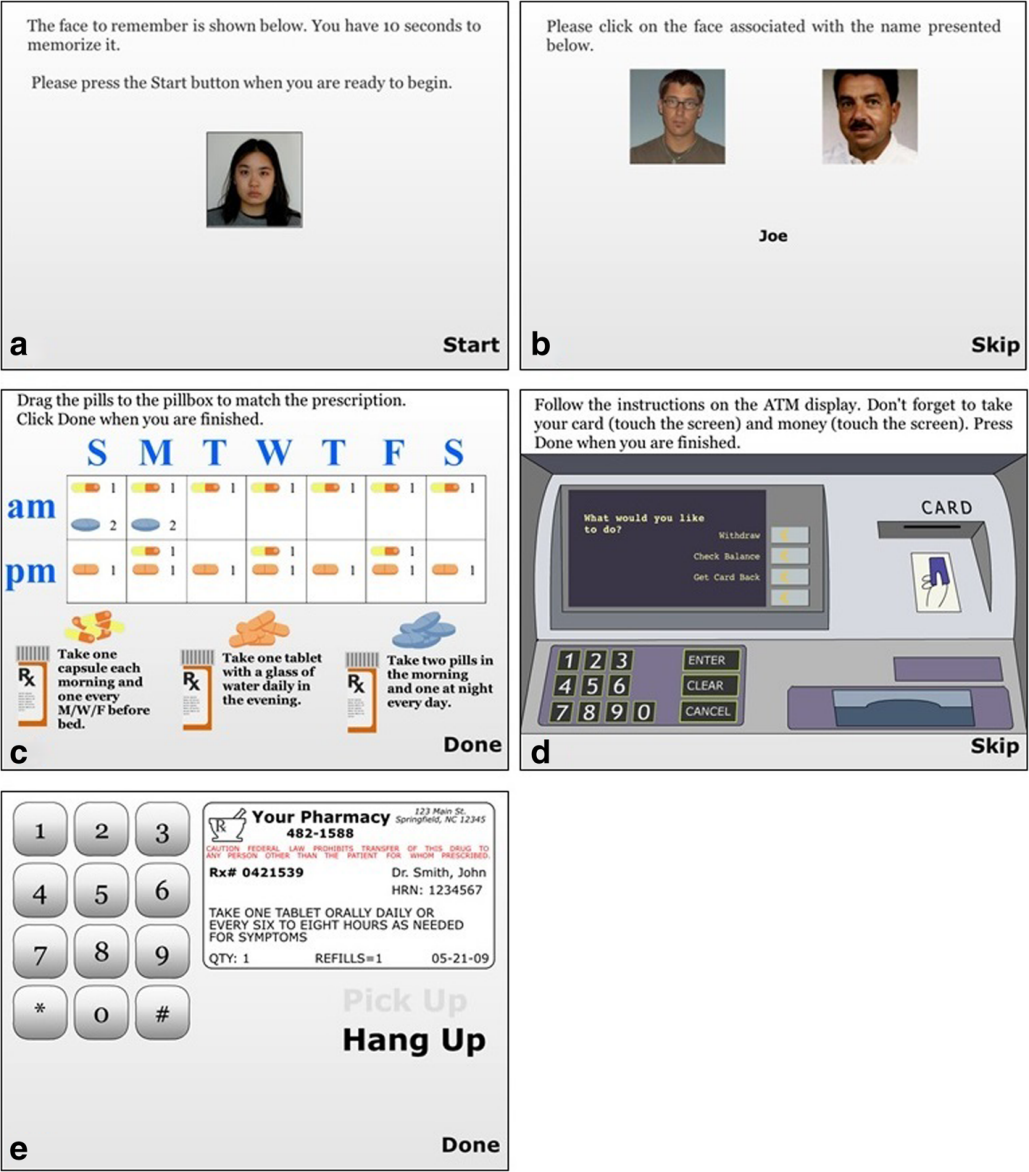
Screenshot of simulation modules in SIMBAC (from left to right and from top to bottom): (**a**) Face Recognition, (**b**) Faces & Names, (**c**) Pillbox, (**d**) ATM, and (**e**) Automated Prescription Renewal

After being instructed in the task, respondents view a digital photo of a human face for 5 s (sex is varied across trials) (Fig. 2a). Next a series of gender- and age-matched novel facial images are presented with the target image and respondents are asked to touch the target image. A practice trial is followed by three successive trials in which the target image is presented with 1, 2, or 3 non-target images.

The face recognition tasks required a collection of photographs of real human faces with similar framing, lighting conditions, and facial expressions across a range of ages, races, and sexes. Rather than collecting or creating new photographs for this, we elected to use the face database described in Minear & Park (2004) [19]. This database provides 580 individual faces, each with the requisite uniformity in framing, lighting, and pose. Each image includes metadata indicating age, sex, and race. Participants in that research signed an informed consent document explaining that the pictures were to be used for psychological research and a legal release form permitting the use of their picture for psychological research. We used the metadata to select specific subsets of images with an appropriate degree of similarity for the targeted level of difficulty.

#### Faces and names

Respondents view a series of facial photos, each paired with a unique name, for 5 s. The next screen presents all the faces viewed in that trial and only one name (Fig. 2b). They must touch the face that was paired with that name. Six trials (3 males, 3 females) of increasing difficulty are presented with 2, 3 or 4 name-face pairs. Images were also selected using the face database described above.

#### Pillbox

On the screen is presented a 7-day pillbox with a.m. and p.m. compartments (14 compartments) and beneath are images of three pill containers with different instructions printed on each label (e.g., “Take one tablet with a glass of water daily in the evening.”). Images of three uniquely shaped or colored tablets/capsules are shown next to each bottle. Participants are instructed to fill the appropriate pillbox by touching and dragging the pills to the correct compartments. (Fig. 2c).

#### Automated teller machine

The initial screen informs respondents they will be asked to withdraw money from an ATM by inserting an ATM card, typing in a Personal Identification Number (PIN), specifying a specific amount of cash and removing their money and ATM card. Next they are told how to insert and withdraw the ATM card and to take the money by touching and dragging the icons. The test screen provides an image of an ATM screen and keypad along with a 4-digit PIN and written instructions to withdraw $40 from their checking account, not to request a receipt and to remember to retrieve their card and money. The screen also shows an ATM card (Fig. 2d). As with real ATMs, respondents can return to the instruction screen by pressing a “Return” button.

#### Automated prescription renewal using a telephone

Respondents are instructed to renew a medication prescription by phone at the pharmacy using the information printed on a pill bottle label and the automated telephone messaging system. The test screen presents an oversized telephone keypad, a label with all necessary information printed on it and an image of a phone receiver (Fig. 2e). Participants touch the phone icon and then hear ringing tone followed by an automated voice recorder with step-by-step instructions.

The phone tree task required reproduction of standard telephone tones indicating a dial tone and the tones associated with each touch-tone phone button. While recordings of these tones are easily found throughout the Internet, we obtained higher quality results by synthesizing the tones directly. To do this, we used ChucK [20], a real-time audio programming language, and wrote small audio snippets to produce exactly the audio waveforms specified by the dual-tone multi-frequency (DTMF) system specifications used by push-button telephones.

The prototypic modules were subsequently tested on computers by members of the research team. Subsequent iterations for improvement took several months to complete. For example, given that the format differs from traditional paper-and-pencil surveys, the decision was made to orient subjects to proper usage of the tablet. An initial screen providing written instructions was added. The next screen instructs them to put their finger on a 2 in. blue oval with “Touch Me!” printed in it. Respondents are presented with 4 trials in which the oval is in different locations. Three additional screens then present a blue circle 1 in. in diameter and a 1”×1” square with instructions to touch the circle and ‘drag’ it across the screen into the square. Specific instructions for each module were also incorporated. After several iterations of improvements, the team decided that the resulting alpha release of the modules was ready to be deployed for field testing.

### Phase 2: Pilot study with 20 older adult volunteers

To evaluate the feasibility of assessing cognition using the modules, twenty community-dwelling, older volunteers were recruited through the Wake Forest Baptist Medical Center, Memory Assessment Clinic and a database of individuals interested in participating in clinical research. The Wake Forest School of Medicine IRB approved the study, and all participants provided written informed consent. All persons were 65 or older and free of dementia. They also completed the MMSE [7], Lawton IADL scale [4], and Katz ADL scale [21]. Participants were given a $40 gift card for each SIMBAC administration.

When completing SIMBAC, participants were seated at a desk in a quiet room next to a trained technician. The modules were presented in the same order on a touch screen computer monitor. Total testing time was approximately 10 min.

After completing the modules, participants were surveyed regarding ease of use, realism and relevance of the modules to their life using a 4-point Likert scale (1 = lowest, 4 = highest). Participants were also asked whether or not they would recommend the use of SIMBAC for assessing cognition. In order to evaluate test-retest reliability, subjects repeated the simulations one month later.

An overall or total scaled performance sum score was calculated for each person. The scoring algorithm used for the entire test ranged from 0 to 5 points and gave a maximum of 1 point (fraction allowed) for each of 5 tasks. The ATM and Automated Prescription Renewal tasks were graded as pass (1)/fail (0). Faces & Names task had a possible raw score of 0/16 to 16/16 depending on the number of trials executed correctly. Face Recognition had a possible score of 0/3 to 3/3 depending on the number of faces recognized. Pillbox had a score of 0/3 to 3/3 depending on how many of 3 pills were placed correctly.

Based on findings from the pilot study, several changes were made to the alpha version of SIMBAC, and important new informatics features were added to the instrument in Phase 2. Instruction pages were modified and user-program interactions streamlined. Crashing of the program, which sometimes occurred with unanticipated click sequences from the user, was addressed as well. The Faces and Faces and Names modules were modified to be less difficult. Improvements in informatics are discussed separately.

The scoring algorithm was also refined to capture a broader range of abilities and ensure uniform assessment of tasks. A SIMBAC Total Accuracy Score (TAS) was devised as the sum of the five module accuracy scores. A score of 2 was awarded for each module completed without any errors, 1 for each module completed with some errors, and 0 points for every module that could not be completed correctly. The updated modules formed the beta version of SIMBAC and were used in Phase 3 of the study.

### Phase 3: Validation of SIMBAC with 162 older adults in a clinical study

A larger sample, consisting of 162 adults over the age of 60 and residing in the local community, was recruited from (a) the Wake Forest Baptist Medical Center, Memory Assessment Clinic and (b) the surrounding metropolitan area with local newspaper and newsletter advertisements. Inclusion criteria included age ≥ 60, English speaking, willing to be retested in 1 year, and consent for a proxy informant to answer questions regarding functional abilities. Exclusion criteria included significant motor, physical, vision, or hearing problems that would prevent testing or use of a computer tablet, current treatment for an acute episode for major depression, bipolar depression, substance abuse or dependence, psychosis or cancer (other than skin cancer), and any of the following diagnoses: severe dementia (defined as MMSE ≤11), Parkinson’s disease, major stroke within past year, or myocardial infarction in the past 6 months. After providing informed consent, participants were administered a comprehensive battery of neurocognitive tests. Unlike in the pilot study, the cognitive status of each participant was determined by a geriatrician (K.M.S., V.W.), who performed a clinical and neuropsychiatric evaluation while blinded to SIMBAC results. Each participant was classified as having No Impairment (NI), Mild Cognitive Impairment (MCI) or Mild Dementia (MD) using National Institute on Aging-Alzheimer’s Association diagnostic criteria [22, 23].

The procedure for administrating the final version of SIMBAC to the participants in Phase 3 was similar to that in Phase 2 except a Motorola XOOM 10.1 in. computer tablet was used. The collection of fine-grained process data was expanded (see informatics section below). The TAS was the primary outcome. Secondary outcome data derived from finer grained data included: subscores based on success/failure in completing a specific step or subtask (coded as 0/1) for each module (e.g., taking receipt from the ATM machine), and time to complete each module. Summary of secondary scores include overall performance in terms of percentage of steps/subtasks completed, and total time score (TTS). Participants also rated the instrument in terms of its realism, reported whether or not they were willing to undergo repeat testing in the future, and indicated whether or not they had completed the simulated activities in the past year.

### Statistical analysis

For Phase 2, we first used descriptive statistics (mean, standard deviation) to examine data collected from the pilot study. Pearson correlation between the cognitive score from SIMBAC and other cognitive measures such as IADL were computed. Two-sample t-tests were used to test whether or not there is a difference in SIMBAC score between the normal and cognitive impaired groups, and effect size was calculated for powering the study in Phase 3. Reliability was assessed using test-retest Pearson correlation. Additionally, descriptive statistics were used to assess the acceptability of the test. For Phase 3, we used both descriptive statistics and bivariate analysis to assess the outcomes. ANOVA was used to test group differences. If an overall significant difference was noted, pairwise comparisons were conducted between the three groups using t tests for continuous measures and Z tests for proportions. All tests were set at the significance level of a = 0.05. When data exhibited non-normality, nonparametric Kruskal-Wallis rank test was used and compared to the parametric test. Effect sizes were calculated using Cohen’s d. As the focus of this article is on development process and informatics lessons learned and the full validation results are reported elsewhere, we only highlight the most important findings regarding validity of the SIMBAC score for Phase 3 in this article. Raw data were processed using R software. All reported statistical analyses were conducted using SAS v.9.3.

### Informatics features

Completion of the pilot study raised awareness of several informatics challenges that needed to be addressed, such as security issues, data management, and overall robustness of the program. Several important informatics features were added to the beta version as a result. The informatics features were important for a larger-scale implementation of the SIMBAC modules.

The security of protected health information (PHI) is a key concern of everyone with access to such data, and the use of mobile devices and third-party servers introduces new risks and vulnerabilities beyond those faced by researchers using traditional assessment methods. For example, mobile devices, by their very mobility, are easy to misplace or accidentally leave somewhere inappropriate. Use of external servers faces the risk of unauthorized access via security vulnerabilities. In each of these cases, there is the potential for exposure of PHI to unauthorized persons.

Though proper encryption can go a long way towards mitigating this risk, we have elected to side step it entirely by eliminating the need to store any PHI on mobile devices. This is accomplished by constructing a unique patient identifier—used only for this one project—that is not derived from any identifying information; we call this the SIMBAC ID, which was then stored in a lookup table that is only saved on secured institutional machines. Each record of the lookup table includes the patient’s medical record number (MRN) and a handful of additional pieces of identifying information. In the instrument itself, the clinician records the SIMBAC ID instead of anything potentially identifying (or that could be inappropriately used for unauthorized identification, such as the MRN). This additional transcription poses the risk of clerical error. We guard against that by also storing sex and age (recorded as 90 for all ages over 89) in the device; absent additional identifiers, these variables alone are insufficient to uniquely identify the patient [24]. When SIMBAC IDs are located in the lookup table, if there are any unmatched records, we can still look for records with the most similar ID, age, and sex.

Interactive computer simulations like SIMBAC offer many opportunities and mechanisms by which to collect detailed data, well in excess of the possibilities offered by traditional assessments. For example, precise timing and detailed records of interaction sequences can be effortlessly captured. However, these fine-grained data are generally not, themselves, particularly useful during analysis, and given the ease with which the simulation can record coarse-grained and synthesized outcome variables, it is tempting to forego collection of seemingly superfluous information. This approach mirrors the limitations inherent to traditional assessments and is conceptually fine as long as the important outcome variables are correctly identified at the outset. However, software is rarely free of bugs, and the fewer distinct variables captured, the more likely it is that a bug that invalidates one of those variables will impair the dataset or even render it entirely useless.

Given this risk, the SIMBAC implementation collects virtually every piece of data available at the time of assessment. The instrument collects enough data to replay a participant’s session, including most taps/touches, drag-and-drop operations, time spent reading instructions, etc. These data are complemented by medium-grained state variables—indications of whether the participant remembered to retrieve the ATM card, for instance—and, of course, synthesized outcome variables summarizing the entire task. With all of these data available, failure to properly record a small number of variables does not impair our ability to analyze the data later: loss of fine-grained data can be tolerated if the more important intermediate and outcome measures are available, and lost outcome measures can be re-created by analyzing the fine-grained traces. Finally, collection of data at this level introduces the ability to synthesize new measures during analysis, well after data collection has concluded.

Regardless of the quantity and type of data collected by the instrument, safeguarding of collected results against either accidental or malicious data loss is essential since, in most cases, lost records cannot be reconstructed. The primary mechanism for guarding against data loss is redundancy [25], and this solution works equally well in the context of mobile device-based research instruments. In SIMBAC, session data are initially stored on the device in a native database form. These data are associated with metadata records that indicate whether each record has been submitted to a centralized collection server. At regular intervals, the instrument attempts to upload via WiFi network all records that have not yet been successfully stored on the central sever. Records that are submitted successfully are simply annotated accordingly on the device; the local device copy of the data is never subject to automatic removal. Further, each record is uploaded to two distinct servers in two distinct formats. To guard against malicious manipulation of local data, each stored record is immutable from within the instrument once the session is complete. Remote data on the collection servers is protected by automatic versioning—retaining all versions of a data record rather than overwriting—and by access control policies that prohibit retrieval, removal, modification, or enumeration of submitted records. Each device is assigned unique, revocable credentials with which to perform uploads. As sessions progress, intermediate results are stored in the local device database so that, should a crash interrupt the session, the administrator can return to the incomplete task, and partial results from all previous tasks are still retained. Finally, a separate backup server in a geographically distinct location performs daily snapshots of the contents of both collection servers while retaining all previous snapshots. Coincidentally, the ability of the app to store data locally facilitates use of the tablet in settings with limited Internet access- data can be stored on the device and will automatically upload to the server when Internet is detected.

While administration of the instrument by trained personnel in controlled laboratory conditions has the potential for significant reduction in the frequency of exceptional events (e.g., software crash, suspending and resuming sessions due to external disruption), it is advantageous for the instrument software to offer sufficient flexibility to cope with such events as they arise. To address this need, the SIMBAC software provides a special “Administration” console that is hidden from participants but which test administrators are trained to access. This console provides basic task navigation functions: skipping ahead, repeating a task, marking an incomplete session as artificially complete, and starting a fresh session altogether. Upon invocation of any of these functions, the administrator is prompted to record a comment documenting the circumstances of the exception. This comment is permanently recorded with the session data. Further, when a task is repeated, all repetitions of that task are recorded and no data is discarded. The administration console also offers panels for viewing, resubmitting, and e-mailing collected data, as well as limited configuration functionality. We also provide a user’s manual to clinicians with comprehensive documentation of the administration console as well as a detailed problem/solution section detailing common problems and the appropriate remedy.

## Results

### Results of phase 2: Pilot study

Analysis of data collected from the pilot study contained two components: (1) evaluation for preliminary evidence of validity, reliability, acceptability, and limitations and (2) assessment of feasibility. Participants were first classified into two groups based on their MMSE score: cognitively impaired (CI; *n* = 10; MMSE =13–25) and cognitively normal (CN; n = 10; MMSE ≥26).

Study groups were comparable in terms of age, gender, race, income and education (*p* > 0.05). Data were lost from 2 participants in the CI group due to an initial computer glitch. The CN group had mean MMSE, IADL, and ADL scores of 29.6, 13.8, and 12, whereas the CI group had mean scores of 19.7, 6.6, and 10.7, respectively (all *p* < .001). The correlation between the SIMBAC performance score and MMSE, Lawton IADL, and Katz ADL were 0.85, 0.76, and 0.45, respectively.

The mean total scaled performance score for SIMBAC was 3.41 for the CN group (SD = 0.98; range 2.23 to 4.69) and 1.49 for the CI group (SD = 0.85; range 0.5 to 2.69) out of a possible 0–5 points. The difference between the two groups is highly significant (p < .001). Nonparametric tests provided similar results. The effect size, which equals 2.09 (Cohen’s d), is substantially larger than the threshold (1.0) for “large effect size” as defined by Cohen’s criterion [26]. The CN group performed significantly better on 5 of 10 simulated tasks at Time 1 and 7 of 10 task parameters at Time 2 (all *p* < 0.05). Table 1 reports the scores for subtasks from individual modules.

**Table 1:** Phase 2: Mean scores of modules across two time points

Computer Module Parameter	Time 1			Time 2		
	CN^a^ (*n* = 10)	CI ^a^ (*n* = 8)	*P*-value^b^	CN (n = 10)	CI (n = 8)	*P*-value
ATM: % PIN correct 1st attempt	38	20	0.41	70	20	*0.02*
ATM: Mean PIN attempts until correct	2	2.1	0.38	1.6	2	0.10
ATM: % successful withdrawals	50	10	0.06	80	20	*0.007*
Faces & Names: Mean total correct (max = 16)	11.25	8.3	*0.006*	11.8	6.7	*0.0001*
Face Recognition: Mean total correct (max = 3)	2.6	1.5	*0.009*	2.7	1.6	*0.003*
Automated Prescription Renewal: % successful dialing 1st attempt	75	50	0.28	60	80	0.33
Automated Prescription Renewal: % successful Rx# on 1st attempt	25	10	0.39	50	0.0	*0.009*
Pillbox: % correct placement (Pill #1)	63	10	*0.02*	80	20	*0.007*
Pillbox: % correct placement (Pill #2)	100	40	*0.007*	80	40	0.070
Pillbox: % correct placement (Pill #3)	86	30	*0.01*	90	50	*0.05*

^a^CN = Cognitively normal; CI = Cognitively impaired

^b^Statistical significant results are shown in boldface

Italicized *p*-value indicates significance at 0.05 level between CN and CI at a given time point

The test-retest correlation coefficient for the total SIMBAC score was 0.73 (*p* < 0.001), suggesting that SIMBAC is reasonably reliable for assessing ecologically relevant cognitive functioning. In the user survey, the average ratings on ease of use, realism, and relevance were respectively 3.0, 3.4, and 2.7 (lowest =1, highest = 4). On the question of whether the participant will recommend the use of SIMBAC for assessing cognition, 19 out of 20 responded positively. Interestingly, Faces & Names received a relatively low score for ease of use (2.5) but a high score for relevance (3.4). Three participants remarked that more time should be given for the Face & Names module. Apparently some of these tasks were too taxing for certain participants.

### Results of phase 3: Validation study

**Fig. 3:**
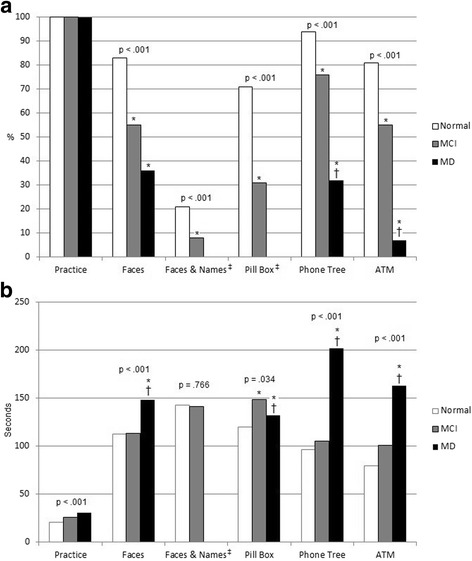
Phase 3: Comparison of completion rates (**a**) and time to completion (**b**); Normal *n* = 78, MCI *n* = 49, Mild Dementia *n* = 28. Reported *p* values represent level of overall test significance. * indicates significant difference, *p* < .05, compared to group with normal cognitive status. † indicates significant difference, p < .05, between MCI and mild dementia subgroups. ‡ These modules could not be completed successfully by any members of the mild dementia subgroup

Preliminary results indicate no loss of data in the validation study. Steps taken to prevent data loss, described in the informatics section, appear to have been successful. Preliminary analysis of 155 participants indicated that all 5 modules reliably differentiated subjects according to cognitive status (all *p* < .001) [27]. Subjects with mild dementia had a lower completion rate than subjects with mild cognitive impairment, whose rate of completion was lower than that of healthy controls (Fig. 3a). Time needed to complete the module also tended to differ between groups with different cognitive status (Fig. 3b). The full results for the instrument, including results from all 162 participants, use the expanded scoring algorithm and measures (TAS, TTS, and error count) and are reported elsewhere [28]. The percentage of persons who completed these activities in the past year is also reported in the main study results, along with subjects’ perception of the realism of the simulation and their willingness to undergo repeat testing if asked to do so by their doctor [28].

## Discussion

We describe the development of a digital, mobile instrument –SIMBAC - and the lessons learned during the development process. SIMBAC was designed to fill a gap in cognitive assessment – i.e., to provide easily interpretable, ecologically valid information about how well an individual can perform relevant, cognitively demanding instrumental tasks. Such information could be useful to individuals themselves, family members, or caregivers and health care providers.

The test-retest correlation coefficient for participants in the pilot study, 0.73, was less than ideal. Modules appear to vary in terms of reliability with some being high and others relatively lower; findings also suggest some tasks may be susceptible to learning effects (e.g. ATM). Learning effects appeared to vary in magnitude across participants between the two testing intervals. What this says about participants is unclear at this point, and a separate investigation into this phenomenon is needed.

Overall, the Phase 2 pilot study established feasibility of SIMBAC and provided preliminary evidence of convergent and criterion validity as well as reliability. The pilot study also revealed many important informatics challenges required to scale up the deployment of SIMBAC for larger and more geographically diverse studies. The Phase 3 validation study, besides demonstrating the validity and reliability of SIMBAC, also demonstrates that regardless of the level of cognitive functioning, participants found SIMBAC modules to be realistic, relevant to their lives and interesting. Many of the informatics challenges were tackled and resolved. The lessons learned, which we describe in detail in the Informatics Features section, should be helpful to researchers and practitioners interested in assessing cognitive and other functions.

This paper contributes to the literature about software development for cognitive assessment. Although recent years have seen the advent of several promising software applications in cognitive assessment, SIMBAC combines useful features in a unique way. Firstly, the only required instrument is a simple, affordable, easy to use handheld device (no expensive or complicated equipment). Secondly, 5 separate modules are included, as opposed to a single situation. A single scenario is more likely to miss important information if an individual performs well in that specific situation but has impairments in other areas. Thirdly, the simulated tasks are highly relevant to the lives of most older adults and offer important insight into real-world functioning. Fourthly, SIMBAC has undergone a four-year period of substantial development and refinement and been tested in two separate studies, one of which featured a relatively large number of participants. Fifthly, satisfactory psychometric properties for SIMBAC have been demonstrated. The presence of these several features concomitantly makes SIMBAC a promising tool for cognitive researchers with potential applications in clinical care.

In the future, SIMBAC may be introduced in the clinical setting. Older adults could easily complete the modules prior to an office visit, the results of which could inform the clinician of any functional limitations the individual may be having and highlight the need for further evaluation. A sustainable implementation plan that includes informatics features that can be scaled up for broad dissemination will need to be tested. Further properties of the program will also need to be explored. For example, future research should determine the correct classification rate of the instrument and optimal scoring thresholds for identifying persons who are likely to be normal or impaired. Necessarily this would require an understanding of how to respond appropriately when a person scores below a certain threshold – what is the next best step? What sort of confirmatory testing is warranted? Also, future research should determine the applicability of the instrument in different cultural contexts and whether or not gender, age, and education are likely to affect results. We welcome collaboration with other researchers who would like to use SIMBAC, and a link to the demo version is included in the declarations section.

The most challenging module for participants was pairing faces with the correct names. Some participants felt overwhelmed when shown several names and faces simultaneously. Consideration is being given to reducing the complexity of the task and recalibrating the scoring algorithm. Still, fatigue was never so severe that it would be expected to interfere with performance.

An interesting variable captured by SIMBAC is the time needed to complete each module. The amount of time taken may have independent prognostic value for detecting cognitive impairment. Measuring accuracy alone could, for example, yield identical scores for two people who successfully withdraw money from an ATM, but require dramatically different amounts of time to do so. Timed assessments of IADLs have demonstrated that needing more time to complete routine activities, a probable impairment in processing speed, contributes to decreased functional capacity [29, 30]. Moreover, slowed performance may be the first indicator of cognitive decline, occurring well before outright inability to perform a given task. The optimal use of time as a factor in evaluating SIMBAC performance has yet to be determined.

There are several limitations of the study. One limitation is that the tasks are limited to activities that can be easily simulated on a computer. More complex but ecologically relevant tasks such as those involving language-based interaction with other individuals or driving a car have not been included. We are currently testing a driving module using virtual reality headsets. Additional studies are needed to test the generalization of performance on SIMBAC to other abilities. A second limitation of SIMBAC is that, while simulation tasks avoid some of the issues that conventional cognitive assessment have – e.g., literacy of the respondents and generally can be more easily implemented in different cultures, specific modules may still need to be modified and adapted to a different cultural environment. For example, the phone-tree may not be immediately applicable to cultures in which prescriptions are not refilled over the phone. On the other hand, because of the visual and generic nature of the video-simulations, modules such as Faces and Faces & Names can be readily implemented in a different culture with minor modifications. SIMBAC may not be ideal for older adults who feel uncomfortable using electronic devices. Users with motor and visual impediments may have difficulty using the tablet.

## Conclusion

In summary, SIMBAC is a low cost, user-friendly, highly versatile tool that can be easily deployed for ecologically valid cognitive assessment. The development process and lessons learned can be used by other health researchers who are interested in developing non-traditional assessment tools using modern informatics and technology.

## Abbreviations

AD: Alzheimer’s Disease
ADL: Activity of Daily Living
ECB: Everyday Cognition Battery
IADL: Instrumental Activity of Daily Living
MCI: Mild Cognitive Impairment
MD: Mild Dementia
MMSE: Mini-Mental Status Examination
SIMBAC: Simulation-Based Assessment of Cognition
TAS: Total Accuracy Score
TTS: Total Time Score
